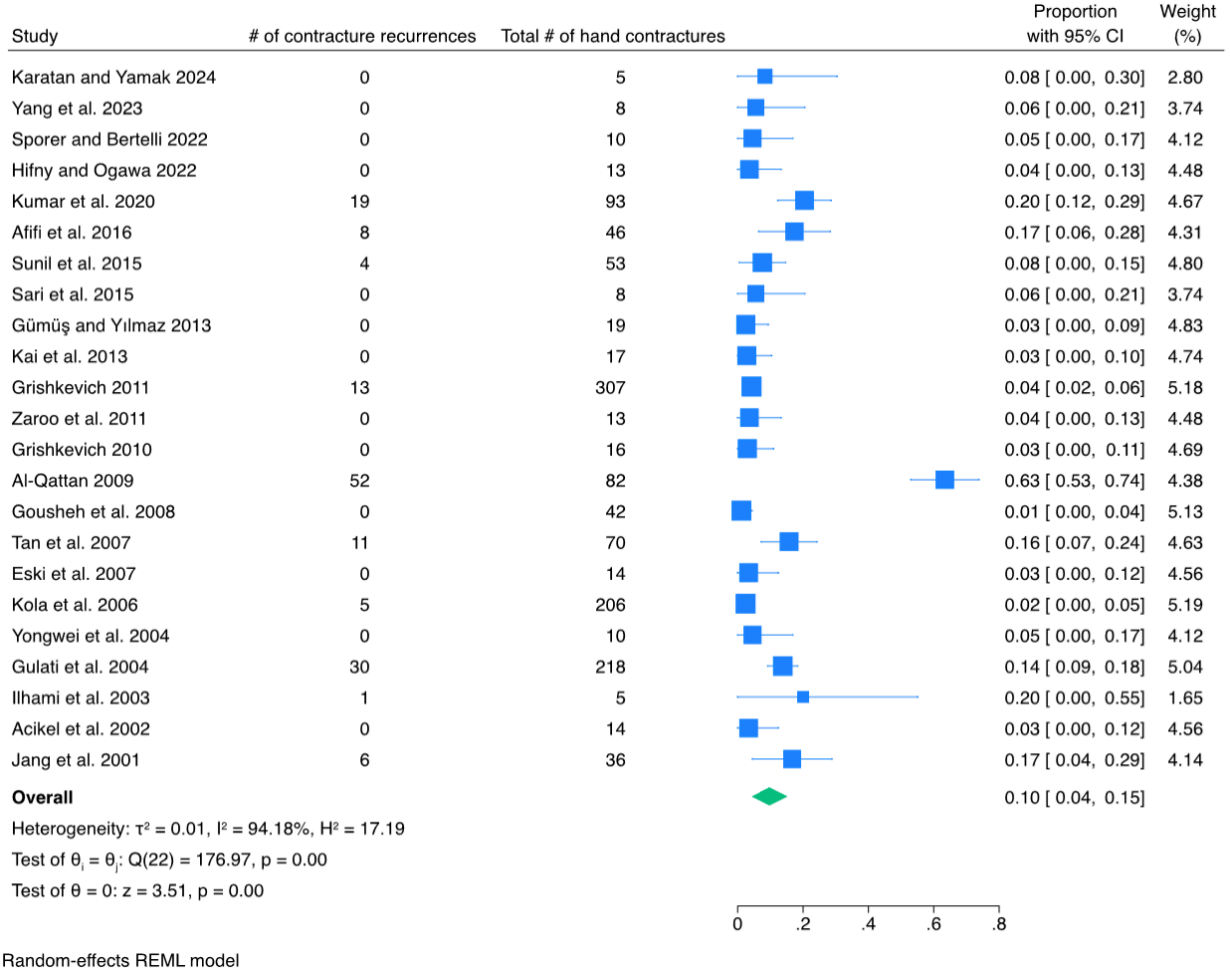# 542 Hand Burn Contracture Recurrence: A Systematic Review and Meta-analysis

**DOI:** 10.1093/jbcr/iraf019.171

**Published:** 2025-04-01

**Authors:** José Arellano, Hilary Liu, Tiffany Jeong, Ana Reis, Justine Kim, Teun Teunis, Francesco Egro

**Affiliations:** University of Pittsburgh Medical Center; University of Pittsburgh Medical Center; University of Pittsburgh Medical Center; University of Pittsburgh Medical Center; University of Pittsburgh Medical Center; University of Pittsburgh Medical Center; University of Pittsburgh Medical Center

## Abstract

**Introduction:**

Deep burn injuries to the hand can result in significant functional limitations. Despite appropriate initial treatment, contractures often develop as these injuries typically heal through scar tissue formation. The unique properties of the skin on the palmar and dorsal aspects of the hand make them variably susceptible to recurrence. Various therapeutic approaches, including skin grafting, Z-plasties, local flaps, regional flaps, island flaps, and free flaps, have been employed to treat post-burn hand contractures.

**Methods:**

A systematic review and meta-analysis was performed and reported according to the Preferred Reporting Items for Systematic Reviews and Meta-Analysis (PRISMA) guidelines. The review protocol was registered on PROSPERO. The study included English-language articles published from 2001 to 2024 that provided extractable data on the incidence of contracture recurrence following surgical intervention for hand burn injuries. Meta-Analysis was performed using a random-effects model.

**Results:**

Among the 2494 articles retrieved, 23 met the inclusion criteria, reporting on 1192 patients (65.8% male, 34.2% female) with 1305 hands that underwent secondary reconstruction for contracture release. These reconstructive burn patients received surgical interventions such as split-thickness skin grafts, full-thickness skin grafts, Z-plasties, and various flaps. The contracture recurrence rate was 0.10 [95% CI: 0.04, 0.15].

**Conclusions:**

Despite various surgical interventions the contracture recurrence rate remains notable at 0.10 [95% CI: 0.04, 0.15]. This underscores the importance of ongoing research and development of more effective treatments to reduce the incidence of contracture recurrence and improve functional outcomes for patients with post-burn hand injuries.

**Applicability of Research to Practice:**

This study highlights that despite various surgical interventions, post-burn hand contractures have a 10% recurrence rate. Clinicians should be aware of this risk when selecting treatments and consider enhanced postoperative care, such as physical therapy and splinting, to improve outcomes. The findings also underscore the need for ongoing research to develop more effective strategies to reduce recurrence and improve functional recovery for burn patients.

**Funding for the Study:**

N/A